# PI3K/Akt and ERK1/2 Signalling Are Involved in Quercetin-Mediated Neuroprotection against Copper-Induced Injury

**DOI:** 10.1155/2020/9834742

**Published:** 2020-07-11

**Authors:** Klara Zubčić, Vedrana Radovanović, Josipa Vlainić, Patrick R. Hof, Nada Oršolić, Goran Šimić, Maja Jazvinšćak Jembrek

**Affiliations:** ^1^Department for Neuroscience, Croatian Institute for Brain Research, University of Zagreb Medical School, 10000 Zagreb, Croatia; ^2^Division of Molecular Medicine, Rudjer Boskovic Institute, 10000 Zagreb, Croatia; ^3^Nash Family Department of Neuroscience, Friedman Brain Institute, and Ronald M. Loeb Center for Alzheimer's Disease, Icahn School of Medicine at Mount Sinai, New York, NY 10029, USA; ^4^Department of Animal Physiology, Faculty of Science, University of Zagreb, 10000 Zagreb, Croatia; ^5^Department of Psychology, Catholic University of Croatia, Ilica 242, 10000 Zagreb, Croatia

## Abstract

Copper, a transition metal with essential cellular functions, exerts neurotoxic effects when present in excess by promoting production of reactive oxygen species (ROS). The aim of the present study was to investigate potential benefits of flavonoid quercetin against copper-induced toxicity. Results obtained with MTT assay indicate that the effects of quercetin are determined by the severity of the toxic insult. In moderately injured P19 neuronal cells, concomitant treatment with 150 *μ*M quercetin improved viability by preventing ROS formation, caspase-3 activation, and chromatin condensation. Western blot analysis revealed that quercetin reduced copper-induced increase in p53 upregulated modulator of apoptosis (PUMA) expression and promoted upregulation of nucleoside diphosphate kinase NME1. Levels of p53 and Bax proteins were not affected by both copper and quercetin. UO126 and wortmannin, inhibitors of ERK1/2 and PI3K/Akt signalling pathways, respectively, prevented neuroprotective effects of quercetin. In severely injured neurons, 30 *μ*M quercetin exerted strong prooxidative action and exacerbated cytotoxic effects of copper, whereas 150 *μ*M quercetin failed to affect neuronal survival. These results demonstrate the dual nature of quercetin action in copper-related neurodegeneration. Hence, they are relevant in the context of considering quercetin as a possible therapeutic for neuroprotection and imply that detailed pharmacological and toxicological studies must be carried out for natural compounds capable of acting both as antioxidants and prooxidants.

## 1. Introduction

Neurodegenerative diseases, such as Alzheimer's disease and Parkinson' disease, are chronic and progressive disorders characterized by slow degeneration of neurons and ultimately cell death. Structural changes disable proper neuronal functioning in affected brain areas and result in the functional deficits characteristic for these diseases [1]. As ageing is the main risk factor for their development, they represent a significant medical and socioeconomic problem worldwide. Despite progress into the understanding of the complex network of molecular and cellular mechanisms that underlie the development of these devastating conditions, no effective pharmacological approaches are yet capable of delaying their onset and slowing down progression [2].

Compelling data suggest that oxidative stress is one of the pivotal factors contributing to the common pathophysiology of neurodegenerative diseases [3, 4]. Oxidative stress occurs when increased accumulation of reactive oxygen species (ROS) overwhelms the brain's intrinsic capacity of oxidative defence, leading to detrimental effects on neuronal functioning and viability. Due to several reasons, including the presence of redox active transition metals, the brain is particularly vulnerable to oxidative injury [5]. Copper is an essential transition metal required for catalytic activity of many brain proteins that are involved in various biological processes, such as respiration, antioxidant activity, catecholamine biosynthesis, and myelin formation [6, 7]. However, if present in excess, unbound copper ions may initiate redox cycling reactions, promote production of ROS, and create an oxidative environment capable of modifying the structure and function of biological macromolecules, thus threatening the functionality and viability of brain cells. In the presence of biological reductants, Cu^2+^ can be reduced to Cu^+^ that via the *Fenton*-catalysed *Haber-Weiss reaction* may drive the generation of extremely toxic hydroxyl radicals, the most dangerous ROS in biological systems [8].

Dysregulation of copper homeostasis, which results in altered redox balance and intracellular redox signalling, is increasingly recognized as an important factor in pathological processes underlying neurodegeneration [9, 10]. Despite some conflicting findings, it seems that serum copper levels are elevated in patients with Alzheimer's disease [11, 12]. Besides a copper-induced increase in ROS generation and the accompanying oxidative stress, aberrant copper/protein interactions may also contribute to copper toxicity and progression of the diseases [8, 13]. Thus, in contrast to healthy conditions, copper binds to amyloid *β* (A*β*) protein specifically in Alzheimer's disease. Cu^2+^ binding to A*β* increases its ability to form ubiquitin-containing aggregates and greatly promotes A*β*-induced generation of ROS [10, 14, 15]. Copper also may compromise the activity of some proteolytic enzymes involved in a degradation of A*β* aggregates. It can directly interact with metal ions essential for the catalytic and structural properties of metalloproteinases, and can confer higher proteolytic resistance to aggregated A*β* species [15]. Furthermore, copper ions promote tau hyperphosphorylation and stimulate its self-aggregation into paired helical filaments *in vitro* [16]. Regarding Parkinson's disease, copper increases *α*-synuclein oligomerization, whereas increased expression of *α*-synuclein enhances neuronal susceptibility to copper toxicity [17]. Copper also prevents the ubiquitination of protein substrates and impairs the proteolytic activity of the ubiquitin-proteasome system, the main pathway for the elimination of the misfolded proteins. Moreover, divalent copper decreases the thermal stability of ubiquitin and promotes its aggregation into spherical particles, probably compromising the neuroprotective function of the ubiquitin-proteasome system [15]. Thus, maintaining copper homeostasis could be considered as an essential prerequisite for the preservation of brain health and proper functioning of neural networks.

Flavonoids represent one of the main groups of bioactive polyphenolic phytochemicals in human diet and are commonly associated with neuroprotection [2, 18]. They can reduce the risk of developing neurodegenerative pathologies, rescue neurons from various forms of toxin-induced neuronal death *in vitro*, suppress neuroinflammation, improve vascular function and cerebral blood flow, and enhance cognitive abilities [2, 19]. The molecular mechanisms underlying the beneficial effects of flavonoids on diverse brain pathologies are primarily attributed to their antioxidant activity (through direct free radical scavenging, induction of endogenous antioxidative defence, and chelation of metal catalysts) and modulation of intracellular signalling pathways involved in neuronal death or survival [2, 18, 20, 21]. In view of their multiple targets and mechanisms of action, various flavonoids became increasingly appreciated as promising candidates for the development of novel and relatively safe therapeutic options in oxidative stress-induced neurodegeneration, particularly in conditions with deregulated metal homeostasis [18, 22].

**Figure 1:**
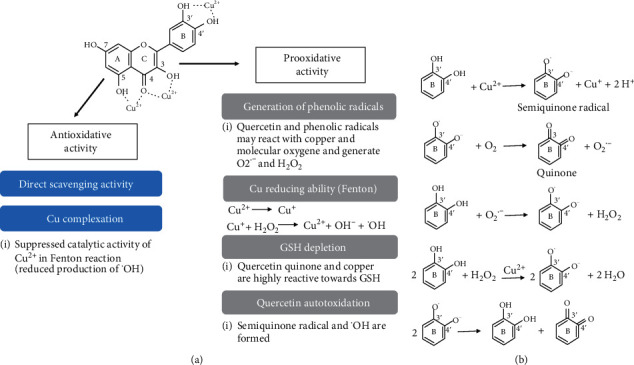
(a) Schematic representation of the antioxidative and prooxidative activities of quercetin. Potential Cu(II) chelating sites of quercetin are shown. Cu complexation may result in antioxidative and prooxidative effects. Antioxidative activity is achieved mainly through the direct scavenging activity and Cu complexation which prevents copper from participating in a Fenton-type reaction. In general, the prooxidative activity of quercetin is mainly exhibited in the presence of transition metals such as copper (simplified chemical reactions of the catechol moiety of quercetin are indicated in (b)). In a reaction of quercetin and Cu^2+^, a quercetin-semiquinone radical is formed together with Cu^+^. The superoxide anion further reacts with quercetin yielding a quercetin-semiquinone radical and hydrogen peroxide. In a reaction with hydrogen peroxide that is catalysed by Cu^2+^, quercetin is oxidized to quercetin-semiquinone radicals that further give quercetin-quinone and quercetin. Hence, these reactions result in redox cycling and further ROS formation. Quercetin also readily autoxidizes generating a semiquinone radical and a hydroxyl radical. Furthermore, the prooxidative ability of quercetin is related to its ability to reduce Cu^2+^. The reduced form (Cu^+^) induces prooxidative reactions. In the Fenton reaction with hydrogen peroxide, a hydroxyl radical is formed. Quercetin semiquinones and quinines facilitate the formation of the superoxide anion and reduce GSH levels further leading to a prooxidative environment.

Quercetin (3,3′,4′,5,7-pentahydroxyflavone) is one of the most abundant natural flavonoids and one of the most potent scavengers of ROS due to its double bonds and hydroxyl groups that stabilize free radicals [23] (Figure 1). Key structural features for scavenging activity are the catechol structure in the B ring, the enol moiety and the 4-keto group in the C ring, and the 5-OH group in the A ring [24].

Similar to other flavonoids, the neuroprotective effects of quercetin against oxidative stress-mediated toxicity have been reported on some types of neuronal cells and assigned to its antioxidant, antiapoptotic, and signal transduction-modifying properties [25–28]. The biological effects of quercetin are also influenced by the presence of metal ions as hydroxy and oxo groups present in the polyphenolic quercetin structure may form complexes with various metal ions [29, 30]. However, although quercetin may inhibit apoptosis and prevent death in some nontumorigenic cells, it may also exert direct proapoptotic effects on tumour cells [31, 32]. Furthermore, depending on the dose, it may exert antioxidative as well as prooxidative effects [33]. Chelation of transition metals is one of the most important mechanisms of the antioxidative action of quercetin. Through metal chelation, quercetin may prevent Fenton's reaction and subsequent ROS generation. In addition, in a stable chelation complex, quercetin exhibits a more powerful antioxidative effect as the scavenging activity of the complex is higher than for quercetin alone [34]. Formation of the complex depends on the metal : ligand ratio and the solvent used. Copper and quercetin may form complexes with copper-to-ligand ratios of 1 : 1, 2 : 1, 1 : 3, and 1 : 2. The 1 : 1 complex is particularly prominent in the presence of excess copper [34]. Metal coordination may occur via a carbonyl oxygen in the C ring and nearby hydroxyl groups (3-OH or 5-OH) in the A or C rings, and between the *ortho*-hydroxyl groups in the B ring [34]. The site between the 5-OH group and the 4-oxo group may be the most important [35]. The estimated binding constant of the irreversible complexation reaction is *K*_1_ = 180 ± 34 × 10^3^ M^−1^ at pH 7.4 [36]. Following complexation, quercetin is rapidly autooxidized and forms quinone radicals and/or quinone intermediates that mediate prooxidative effects. The other mechanism that contributes to the prooxidative activity of quercetin is its ability to reduce divalent copper to Cu(I). Thus, the cytotoxic effects of quercetin may be explained not only by the increase in ROS production but also by the production of highly oxidant and electrophilic oxidation products [29, 36]. A schematic representation of the antioxidative and prooxidative activities of quercetin is shown in Figure 1.

In this context, the aim of the present study was to investigate the effects of quercetin against copper-induced toxicity in P19 neurons to understand better the mechanistic interactions between, and cellular response to, concomitant treatment with natural antioxidants and potentially toxic metals. Ultimately, this might be useful for the improvement of therapeutic options in copper-driven neurodegenerative changes.

## 2. Materials and Methods

### 2.1. Chemicals

All-*trans*-retinoic acid (ATRA), cytosine-arabinofuranoside (AraC), 1,4-diamino-2,3-dicyano-1,4-bis(2-aminophenylthio)-butadiene (UO126), 2′,7′-dichlorofluorescin diacetate (DCF-DA), 3-(4,5-dimethylthiazol-2yl)2,5-dyphenyl-2H-tetrazolium bromide (MTT), and Hoechst 33342 were purchased from Sigma-Aldrich (St. Louis, MO, USA). All chemicals used for the maintenance and differentiation of P19 cells, including culture medium (Dulbecco' Modified Eagle's Medium, DMEM), foetal bovine serum (FBS), trypsin, antibiotics, L-glutamine, and an insulin-transferrin-selenium-ethanolamine (ITS-X) supplement were purchased from Sigma-Aldrich (St. Louis, MO, USA) or Gibco (Paisley, UK). Wortmannin was purchased from Ascent Scientific (Princeton, NJ, USA). Copper sulfate pentahydrate was obtained from Kemika (Zagreb, Croatia). All other chemicals used were of analytical grade.

### 2.2. P19 Cell Culturing and P19 Neuronal Differentiation

Undifferentiated P19 cells (a pluripotent mouse embryonal carcinoma cell line) were kindly provided by J. Pachernik (Prague, Czech Republic). They were cultured in high-glucose DMEM containing 10% heat-inactivated FBS, 2 mM L-glutamine, 100 units/ml penicillin G, and 100 *μ*g/ml streptomycin (growth medium) in a humidified atmosphere of 5% CO_2_ at 37°C. They were passaged every two days.

Neuronal differentiation of P19 cells was induced by exposure to 1 *μ*M all-*trans*-retinoic acid (ATRA) for 4 days. Exponentially growing P19 cells (1 × 10^6^) were seeded into 10 cm bacteriological grade Petri dishes containing 10 ml of DMEM medium supplemented with a reduced concentration of FBS (5%), 2 mM L-glutamine, and antibiotics (induction medium). ATRA is added immediately after plating. Aggregates (embryonic bodies) of P19 cells were formed after 1-2 days. After 48 h, the old medium was replaced with the fresh ATRA-containing medium and aggregated cultures were grown for an additional two days.

After the 4-day ATRA treatment, P19 embryonic bodies were harvested, washed with phosphate-buffered saline (PBS), dissociated by trypsinization and pipetting, collected by centrifugation (1,250 rpm, 5 min), and finally resuspended in growth medium. For optimal neuronal differentiation, single cells at a density 10^5^ cells/cm^2^ were plated onto 96-well plates or 35 mm Petri culture dishes (Sarstedt, Newton, NC, USA and Nunc A/S, Roskilde, Denmark) and grown in growth medium for two days. Finally, the growth medium was changed to serum-free medium containing DMEM supplemented with an ITS-X solution containing insulin, transferrin, selenium and ethanolamine solution (Gibco), 0.5 mM L-glutamine, and antibiotics (neuron-specific medium) and cells were grown for 2 more days in the presence of 10 *μ*M of the mitotic inhibitor AraC to inhibit proliferation of nonneuronal cells. Complete neuronal differentiation was confirmed with monoclonal anti-tubulin *β*-III mouse IgG, clone TU-20 conjugated with Alexa Fluor 488 (Millipore, Temecula, CA), see Supplementary Material, Figure S1. At the end of differentiation procedure, P19 neurons exert morphological, neurochemical, and electrophysiological properties resembling neurons from the mammalian brain, and represent an established model for pharmacological studies [25, 26, 37].

### 2.3. Drug Treatment

In all experiments, P19 neurons were treated for 8 days from the initiation of differentiation (DIV8). Each batch of cultured cells was divided into control and drug-treated groups. For inducing oxidative damage, P19 neurons were incubated with 0.5 or 1 mM CuSO_4_ in the neuron-specific medium for 24 hours, alone or in the presence of various concentrations of quercetin. All selected concentrations of quercetin did not modify the viability of P19 neurons when applied alone [25, 38]. To examine the effects of quercetin on the activation of the signalling pathways of phosphatidylinositol-3-kinase (PI3K)/Akt and extracellular signal-regulated kinases 1/2 (ERK1/2), P19 neurons were pretreated with wortmannin and UO126, respectively. Wortmannin is a nonspecific, covalent inhibitor of PI3K, an upstream activator of Akt. Wortmannin (30 nM) and UO126 (10 *μ*M) were present 60 min prior and during the exposure to 0.5 mM copper and 150 *μ*M quercetin. The concentrations were chosen based on our preliminary experiments. We selected the smallest concentrations of inhibitors that modified the neuroprotective effect of quercetin but did not affect neuronal viability when applied alone.

### 2.4. Assessment of Neuronal Viability

Survival of P19 neurons was assessed by using colorimetric MTT assay. Cell viability was estimated according to the ability of P19 neurons to cleave MTT to an insoluble formazan product due to the activity of mitochondrial dehydrogenases. At the end of the incubation period, 40 *μ*l of MTT solution prepared in neuron-specific medium (final concentration 0.5 mg/ml) was added to each well and incubated for 3 h at 37°C. Precipitated formazan was dissolved by adding 160 *μ*l of dimethyl sulfoxide (DMSO). The absorbance of each well was recorded by an automatic microplate reader at 570 nm. After blank subtraction from all absorbance readings, cell survival was expressed as the percentage of absorbance of quercetin- and copper-treated cells relative to that of untreated control cells.

### 2.5. Measurement of Intracellular ROS Accumulation

Intracellular production of ROS as a measure of cellular oxidative stress was detected using the cell permeable substrate DCF-DA. This fluorogenic probe is widely used for monitoring cellular oxidative activity. DCF-DA diffuses into the cells and becomes hydrolysed to nonfluorescent 2′,7′-dichlorofluorescin by intracellular esterases. Accumulated 2′,7′-dichlorofluorescin reacts with intracellular ROS and forms the highly fluorescent compound dichlorofluorescein (DCF). The method is reliable and efficient for evaluating the potency of prooxidants as well as the efficacy of antioxidants against oxidative stress and is suitable as a marker for the total ROS production [39]. Following treatment, P19 neurons were incubated with 100 *μ*M DCF-DA in PBS for 1 h in darkness, rinsed, and incubated for an additional 1 h in PBS. Oxidation of DCF-DA to DCF was determined using the Varian Cary Eclipse Fluorescence Spectrophotometer (Varian Optical Spectroscopy Instruments, Mulgrave, Victoria, Australia) with an excitation wavelength (*λ*_ex_) of 504 nm and an emission wavelength (*λ*_em_) of 529 nm. The results were expressed as the percentage of fluorescence intensity obtained in the control group.

### 2.6. Determination of Reduced Glutathione (GSH) Levels

GSH is one of the major nonenzymatic antioxidants present in neuronal cells. We monitored changes in GSH levels with the luminescence-based GSH-Glo Glutathione Assay (Promega, Madison, WI, USA) according to the manufacturer's instructions. Briefly, the assay couples two chemical reactions. The first reaction is the conversion of a luciferin derivative into luciferin, which is catalysed by glutathione-S-transferase. In the second reaction catalysed by firefly luciferase, the amount of luciferin produced is detected as a luminescent signal. The intensity of the signal is proportional to the GSH content. At the end of exposure to copper and quercetin, the medium was removed and 100 *μ*l of the GSH-Glo reagent was added in each well. The samples were incubated for 30 min at RT. Thereafter, 100 *μ*l of the luciferin detection reagent was added to each well, mixed briefly, and incubated for a further 15 minutes. Emitted light was quantified by the Fluoroskan Ascent FL luminometer (Thermo Fisher Scientific).

### 2.7. Determination of Caspase-3/7 Activity

Caspase-3/7 activity was monitored by using the Apo-ONE Homogeneous Caspase-3/7 Assay (Promega, Madison, WI, USA). These two members of the caspase family are the key effector enzymes in caspase-dependent apoptosis. To perform the assay, the cell permeabilization buffer and nonfluorescent caspase substrate Z-DEVD-rhodamine 110 were mixed (50 *μ*l) and added directly to P19 neurons treated with quercetin and copper in 50 *μ*l of culture medium. The sample was shaken for 5 min and incubated at RT in the dark for 4 h. Cleavage of the DEVD peptides by activated caspase-3/7 releases rhodamine 110, which becomes intensely fluorescent after excitation. The amount of generated fluorescent product is proportional to the amount of caspase-3/7 cleavage activity in each sample. The emitted fluorescence was recorded on a plate reader (Fluoroskan Ascent FL, Thermo Fisher Scientific) at an excitation wavelength of 485 nm and an emission wavelength of 538 nm.

### 2.8. Determination of Nuclear Changes by Hoechst Staining

Hoechst 33342 staining was used to examine nuclear condensation in P19 neurons exposed to copper and quercetin. Following treatment, attached and floating cells were collected, centrifuged at 250 g, resuspended in 50 *μ*l of culture medium, and stained with 5 *μ*M Hoechst 33342 for 10 min at RT. Cells were analyzed under a fluorescent microscope. Cells with bright blue nuclei were counted. The percentage of cells with condensed nuclei in relation to the total number of cells was determined by counting at least 500 neurons.

### 2.9. Western Blot Analysis of p53, Bax, PUMA, and NME1/2 Protein Expression

The expression of proteins was analyzed in total cell extracts. Following treatment, P19 neurons grown in 25 cm^2^ flasks were washed twice with PBS, lysed by scrapping into 100 *μ*l of RIPA buffer (Sigma-Aldrich, St. Louis, MO, USA) containing protease inhibitors (cOmplete, Mini, EDTA-free Protease Inhibitor Cocktail Tablets; Roche, Indianapolis, IN, USA), and sonicated. The protein concentration was determined using the Pierce BCA Protein Assay Kit (Thermo Fisher Scientific, Waltham, MA, USA). A standard curve was developed using a series of bovine serum albumin dilutions in the 100 *μ*g/ml to 1500 *μ*g/ml range, and a linear plot of concentration vs. absorbance (at 570 nm) was generated to determine protein concentration in different samples. Extracted proteins were resolved using SDS-PAGE and analyzed by immunoblotting on nitrocellulose membranes (Whatman, GE Healthcare Life Sciences, Berlin, Germany). Nonspecific binding was blocked by 5% nonfat milk in Tris-buffered saline containing 0.05% Tween 20 (TBST) for 1 h at RT. Immunoblots were incubated overnight with a primary antibody and then for 2 h at RT by an appropriate secondary antibody. Blots were incubated with the primary anti-p53 antibody (clone Pab 1801: sc-98 and Pab 240: sc-99, Santa Cruz Biotechnology, 1 : 500; clone Pab 421, kindly provided by Prof. J.-C. Bourdon, Dundee, United Kingdom, 1 : 200), anti-PUMA*α*/*β* antibody (G-3, sc-374223, Santa Cruz Biotechnology, 1 : 500), anti-Bax antibody (B-9: sc-7480, Santa Cruz Biotechnology, 1 : 500), and anti-NME1/NME2 antibody (kindly provided by Drs. I. Lascu, Bordeaux, France, and S. Volarević, Rijeka, Croatia, 1 : 3000). The anti-*β*-actin antibody (AC-15, Sigma-Aldrich, 1 : 5000) was used for normalization. Immunoreactive bands were visualized by chemiluminescence detection (Western Lightning Plus-ECL, Enhanced Chemiluminescence Substrate, PerkinElmer, Waltham, MA, USA) of an HRP-labelled secondary antibody (anti-mouse IgG, GE Healthcare, ECL mouse IgG, 1 : 5000) and anti-rabbit IgG (Cell Signaling Technology, 1 : 3000) and quantified by using ImageJ NIH software after detection with Alliance 4.7 (UVItec Cambridge, London, UK).

### 2.10. Statistical Analysis

Statistical analyses of the data were carried out using GraphPad Software (San Diego, CA). All values are represented as mean ± standard deviation (SD). Comparisons between group means were evaluated by one-way ANOVA, and when statistically significant, post hoc analysis with Dunnett's multiple comparison test or Tukey's test followed. Data on samples fit assumptions for ANOVA (dependent variable is continuous, observations are independent, there are no significant outliers, dependent variable is approximately normally distributed for each of the independent variables, and independent variable combinations pass Levene's test for homogeneity of variances). The statistical significance level was set at *α* = 0.05.

## 3. Results

### 3.1. Effects of Copper and Quercetin on Viability of P19 Neurons

**Figure 2:**
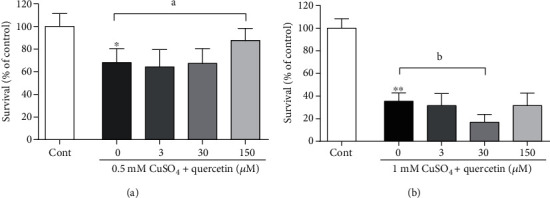
Effects of quercetin on the survival of P19 neurons in the presence of excess copper. For the analysis of neuronal survival, P19 neurons (30 × 10^3^/well) were exposed to the indicated concentrations of CuSO_4_ and quercetin in a 96-well plate for 24 h, and for 3 h with MTT afterwards. Precipitated formazan crystals were dissolved with DMSO, and the absorbance was recorded at 570 nm. In moderately injured neurons exposed to 0.5 mM CuSO_4_, 150 *μ*M quercetin improved neuronal survival (a). Treatment with 1 mM CuSO_4_ induced more severe neuronal death. The presence of 30 *μ*M quercetin further exacerbated the cytotoxic effects of copper, whereas a 150 *μ*M concentration was without effect (b). Data represent the mean ± SD from four independent experiments performed in quadruplets. ^∗^*P* < 0.0001 vs. cont (0 group); ^a^*P* < 0.05 and ^b^*P* < 0.01 vs. the copper-treated group (one-way ANOVA followed by Tukey's test: (a) *F*_(4, 39)_ = 101.1, *P* < 0.0001; (b) *F*_(4, 31)_ = 15.04, *P* < 0.0001).

As previously published [40], exposure to increasing concentrations of copper reduced the viability of P19 neurons. Treatment with 0.5 mM CuSO_4_ for 24 h induced moderate cell death (neuronal viability was decreased by 35%), whereas exposure to 1 mM CuSO_4_ induced more pronounced reduction of neuronal survival and approximately doubled the number of dead cells. We investigated the effects of three different concentrations of quercetin (3, 30, and 150 *μ*M) on copper-induced neuronal death. These concentrations of quercetin did not induce apparent changes in neuronal viability when applied alone [38]. As presented in Figure 2(a), in P19 neurons simultaneously exposed to 0.5 mM CuSO_4_ and quercetin, the highest concentration of quercetin exerted a neuroprotective effect and improved neuronal survival, although neuronal viability was not completely returned to the control value of the vehicle-treated group (87.5% of the control group, *P* < 0.05). Conversely, when quercetin was applied in the presence of 1 mM CuSO_4_, a decrease in neuronal survival was obtained with 30 *μ*M quercetin (neuronal viability was further decreased by 48% in comparison with the group exposed solely to 1 mM CuSO_4_), while treatment with 3 or 150 *μ*M quercetin did not modify the effects of copper (Figure 2(b)).

### 3.2. The effects of Quercetin against Copper-Induced Neuronal Death Are Related to ROS Generation

**Figure 3:**
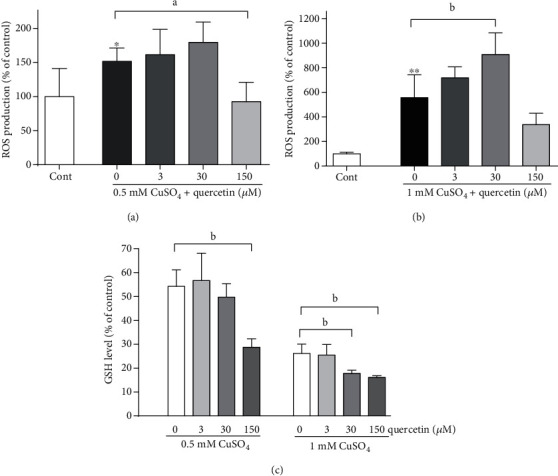
Effects of quercetin on ROS production and GSH levels in copper-mediated oxidative stress conditions. ROS levels were quantified by measuring fluorescence intensity after incubation with 2′,7′-dichlorofluorescin diacetate (DCF-DA). In P19 neurons exposed to 0.5 mM CuSO_4_, only the highest concentration of quercetin prevented a copper-induced increase in ROS production (a). In severely damaged neurons, 30 *μ*M quercetin exacerbated a prooxidative effect of copper (b). 30 and 150 *μ*M quercetin exacerbated a copper-induced decrease in GSH content (c). Data represent the mean ± SD from four independent experiments performed in quadruplets. ^a^*P* < 0.05 and ^b^*P* < 0.01 vs. the copper-treated group; ^∗^*P* < 0.05, ^∗∗^*P* < 0.0001 vs. control (one-way ANOVA followed by post hoc Dunnett's test).

Copper ions upregulated intracellular ROS generation in a dose-dependent manner (^∗^*P* < 0.05 and ^∗∗^*P* < 0.0001 vs. control; one-way ANOVA followed by Dunnett's test, Figure 3). In mild oxidative conditions induced by exposure to 0.5 mM CuSO_4_, 3 and 30 *μ*M quercetin did not affect ROS amounts, while the presence of 150 *μ*M quercetin preserved ROS production at the physiological levels, indicating an antioxidative mode of quercetin action at this concentration (Figure 3(a)). In a highly oxidative environment (Figure 3(b)), 3 and 150 *μ*M concentrations were without effects, whereas 30 *μ*M quercetin upregulated ROS production (62.8%, *P* < 0.01), suggesting a prooxidative mechanism of its action. Interestingly, in severe oxidative stress, 150 *μ*M quercetin also tended to decrease ROS production. It reduced ROS levels by 39.6% in comparison to copper-treated cells (from 557.9 to 336.9 of relative fluorescence units (RFU)), although this reduction was not statistically significant. Similarly, a small although insignificant increase of ROS (18.2%, from 151.9 to 179.6 RFU) was observed for 30 *μ*M quercetin in mild oxidative conditions.

Alterations in ROS production may affect levels of GSH, the most important endogenous mechanism of nonenzymatic antioxidative defence. As previously reported, exposure to divalent copper ions depleted GSH content in neuronal cells in a dose-dependent manner [40]. In this study, 150 *μ*M quercetin induced a further decrease in GSH levels in mild oxidative stress conditions, whereas in P19 neurons treated with 1 mM CuSO_4_, the GSH-reducing effect was observed for 30 and 150 *μ*M concentrations (Figure 3(c)).

### 3.3. Quercetin Prevents Copper-Induced Changes in Chromatin Condensation and Decreased Caspase-3/7 Activity

**Figure 4:**
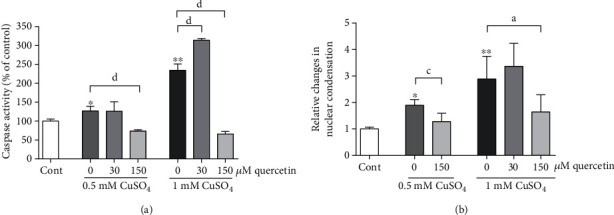
Effects of quercetin on caspase-3/7 activity and nuclear condensation. In moderately injured P19 neurons, a protective concentration of quercetin abolished copper-induced caspase-3/7 activation (a) and nuclear condensation (b), while in severely injured neurons, a prooxidative concentration of quercetin increased caspase activation without affecting chromatin condensation. Results of caspase-3/7 activity are expressed as mean ± SD from 4 independent experiments performed in duplicates. Changes in nuclear condensation were determined from 3 independent experiments. At least 500 nuclei per group were examined in each experiment, in randomly selected fields. ^a^*P* < 0.05, ^c^*P* < 0.001, and ^d^*P* < 0.0001 vs. the copper-treated group; ^∗^*P* < 0.01 and ^∗∗^*P* < 0.0001 vs. control (one-way ANOVA followed by post hoc Dunnett's test).

ROS may be an initiating factor in triggering caspase-dependent and caspase-independent neuronal deaths that are both characterized by nuclear changes in the form of chromatin condensation [41]. However, the effects of flavonoids on cell viability may also result from the inhibition of the activity of the executioner caspases-3/7 in the classical apoptotic pathway or may be independent on caspase activity [25, 42]. To distinguish between these two possibilities, we monitored caspase-3/7 activity and chromatin density. As shown in Figure 4(a), in P19 neurons exposed to both 0.5 mM and 1 mM CuSO_4_, 150 *μ*M quercetin completely prevented a copper-induced increase in caspase-3/7 activation. In mild oxidative stress, 30 *μ*M quercetin did not modify caspase-3/7 activity, while in a highly oxidative environment, this concentration of quercetin further exacerbated copper-induced enhancement of caspase-3/7 activation. The intensity of chromatin condensation was evaluated by staining with the fluorescent DNA dye Hoechst 33342. In copper-treated P19 neurons, the intensity of DNA staining with Hoechst 33342 was significantly increased. The number of nuclei with condensed chromatin was increased by 90% and 189% following exposure to 0.5 mM CuSO_4_ and 1 mM CuSO_4_, respectively, and this effect on the increase in chromatin density was more pronounced than the effect on caspase-3/7 activity. Concomitant treatment with 150 *μ*M quercetin completely prevented changes of chromatin morphology in both copper-treated groups, whereas exposure to 30 *μ*M quercetin and 1 mM CuSO_4_ did not further promote copper-induced changes in chromatin condensation (Figure 4(b)).

### 3.4. Quercetin Prevents Copper-Induced Upregulation of PUMA Expression and Upregulates Expression of NME1 Protein

**Figure 5:**
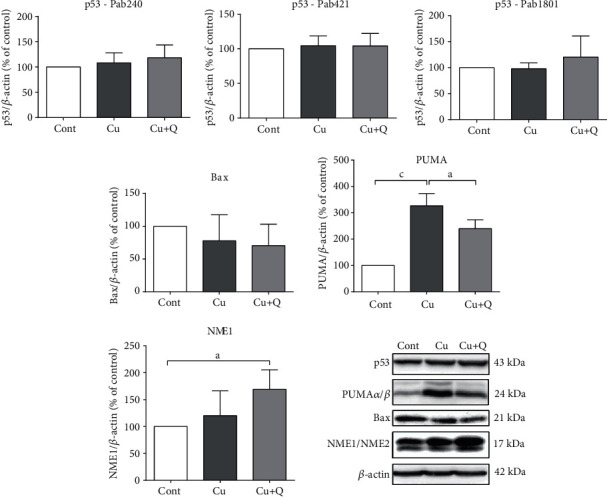
Signalling by apoptotic and oxidative stress-related pathways. The effects of copper and quercetin on the expression of p53, Bax, PUMA*α*/*β*, and NME1/NME2 in moderate oxidative stress were monitored for 24 h from the beginning of treatment with 0.5 mM CuSO_4_ and 150 *μ*M quercetin, and proteins of total cell extracts were separated on 10 or 11% SDS polyacrylamide gel electrophoresis and transferred to a nitrocellulose membrane. Blots were probed with primary antibodies, followed by the appropriate horseradish peroxidase-labelled secondary antibody. Immunoreactivity was detected using enhanced chemiluminescence. *β*-Actin was used as loading control for normalization. Data are expressed as mean ± SD from up to five immunoblots of three independently prepared cell lysates. Exposure to copper and quercetin did not affect p53 and Bax expression. However, quercetin diminished copper-induced upregulation of PUMA and stimulated NME1 expression. Following densitometric analysis, obtained data were analyzed with one-way ANOVA followed by Dunnett's test (^a^*P* < 0.05, ^c^*P* < 0.001). Representative Western blots are also presented.

To elucidate the molecular mechanisms of the neuroprotective action of quercetin further, we monitored changes in the expression of proteins involved in the regulation of neuronal death cascade and whose expression is typically modified in oxidative conditions. As quercetin at 3 and 30 *μ*M concentrations did not modify neuronal survival and ROS formation, we assessed changes induced only by 150 *μ*M quercetin. Transcription factor p53 plays an important role in determining neuronal death or survival by coordinating transcription of pro- and antioxidative proteins and through the regulation of mitochondrial metabolism and ROS production [43]. However, by using three different p53 antibodies, we found no copper- or quercetin-induced changes in p53 levels. The same result was obtained for Bax, a downstream target of p53. However, expression of PUMA, an important regulator of neuronal apoptosis, was highly upregulated by 0.5 mM CuSO_4_. This increase in PUMA expression was reduced in P19 neurons concomitantly treated with quercetin. We also monitored changes in the expression of nucleoside diphosphate kinase NME1. In response to environmental stimuli, this enzyme can translocate to the nucleus and regulate transcription and DNA repair [44]. Although expression of NME1 protein was not significantly altered in P19 neurons treated with 0.5 mM CuSO_4_ alone, NME1 gene expression was enhanced by 69% in the presence of 150 *μ*M quercetin (Figure 5).

### 3.5. The Neuroprotective Effect of Quercetin Is Mediated through the Modulation of Akt and ERK1/2 Signalling Pathways

**Figure 6:**
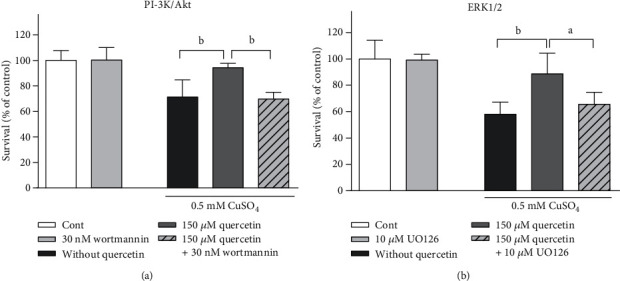
Effects of wortmannin and UO126 against the neuroprotective action of quercetin in moderately injured P19 neurons. P19 neurons were treated with 30 nM wortmannin and 10 *μ*M UO126 for 1 h prior to and during the treatment with 0.5 mM CuSO_4_. At 24 h from the beginning of treatment with copper and quercetin, the survival of P19 neurons was determined by MTT assay. Exposure to both wortmannin (a) and UO126 (b) prevented the beneficial effects of quercetin on neuronal survival in copper-induced oxidative stress conditions. Values are expressed as means ± SD from five independent experiments performed in quadruplets. ^a^*P* < 0.05 and ^b^*P* < 0.01 vs. the copper- or the copper- and quercetin-treated group (one-way ANOVA followed by post hoc Tukey's test).

To investigate whether neuroprotective effects were mediated through the modulation of intracellular signalling, we treated P19 neurons concomitantly with copper, quercetin, and inhibitors of the Akt and ERK signalling pathways. Wortmannin is a specific, covalent inhibitor of PI3K. Akt/PKB is located downstream of PI3K and, therefore, functions as part of a wortmannin-sensitive signalling pathway. UO126 is a highly selective inhibitor of mitogen-activated protein kinase kinase MEK1/2 that further activates ERK1/2 by phosphorylation at Thr and Tyr residues [45]. Our results indicate that the neuroprotective effect of quercetin was mediated through the activation of Akt and ERK1/2 signalling as the prosurvival effect of quercetin was no longer visible in the presence of wortmannin (Figure 6(a)) and UO126 (Figure 6(b)).

## 4. Discussion

Deregulation of metal homeostasis, accompanied by enhanced production of free radicals and increased oxidative stress, is directly involved in the onset and progression of neurodegenerative diseases (for reviews, see [13, 46]). Natural antioxidants have potential to reestablish redox homeostasis and reduce or prevent metal-induced oxidative damage [47–49]. Besides their ability to act as antioxidants, metal chelators, and modulators of intracellular signalling, flavonoids may inhibit the formation of A*β* fibrils [50, 51] and improve the activity of the ubiquitin-proteasome system [52, 53]. Hence, flavonoids, including quercetin, represent a promising multitarget drug option, particularly if applied during the early stage of neurodegenerative processes. Accordingly, a few dietary intervention studies have demonstrated an ability of phytochemicals to improve neurological dysfunction [54]. We studied the effects of quercetin, one of the most abundant flavonoids from human diet, against copper-initiated neuronal death. Previous studies indicated that Cu^2+^-induced oxidative injury can be attenuated by polyphenols [6, 55]. Our results suggest that the effects of copper and quercetin on neuronal survival could be governed by their concentrations and are dependent on the severity of toxic insult. In mild oxidative stress, quercetin may exert a desirable neuroprotective action and improve neuronal survival. However, in severely damaged neurons, quercetin, applied at specific dose, is capable of exacerbating the toxic effect of copper leading to more pronounced neuronal death. The dual effects of quercetin on cell viability were also observed in other studies [33, 56].

The toxic effects of copper are mostly attributed to its role in oxidative stress induction and downstream death events [27, 57]. Hence, the prevention of ROS production might be a promising strategy against copper-induced toxicity and neurodegeneration in general [58]. We previously demonstrated that quercetin applied alone attenuated the generation of radical species in P19 neurons [38]. However, monitoring ROS production in the presence of copper ions revealed the antioxidative as well as prooxidative effects of the polyphenolic structure of quercetin, in a manner related to its effects on neuronal viability. In copper-induced mild oxidative stress, the neuroprotection offered by quercetin was associated with its antioxidative properties, while its prooxidative ability largely contributed to increasing neuronal death in severe oxidative stress. In P19 neurons exposed to low and high concentrations of hydrogen peroxide, quercetin exhibited only an antioxidative action [25], pointing out to the contribution of copper ions in its prooxidative action.

Additional studies also demonstrated that the effects of quercetin in the presence of copper ions are dose-dependent and related to the intensity of oxidative processes [59–61]. They also pointed out that the mechanisms of quercetin/copper interactions are rather complex and worth studying further. It has been shown that quercetin induces oxidative DNA damage at specific sites in the presence of Cu(II); however, with high doses of quercetin, DNA damage is decreased [59]. In another study, quercetin at low Cu^2+^ concentrations protected from DNA damage, whereas quercetin at higher Cu^2+^ concentrations promoted DNA cleavage [60]. By measuring the extent of lipid peroxidation on low-density lipoprotein preparations, Filipe et al. [61] reported that quercetin at high Cu^2+^ concentrations decreased lipid peroxidation. In the presence of low Cu^2+^ concentrations, quercetin exhibited a dual effect acting as a prooxidant at low concentration and exerting antioxidant activity when applied at high concentrations. Flavonoids exert their antioxidative action via various mechanisms including direct ROS trapping, prevention of peroxidation, inhibition of ROS-producing enzymes, and chelation of transition metals [34]. Regarding the chelation process, interactions between quercetin and copper(II) are promoted in the presence of excess copper, and the copper-quercetin complex is a better free radical acceptor than quercetin alone [30, 34]. However, in the presence of metal ions, natural antioxidants may switch their biological effects to prooxidant activity. Several studies investigating the interactions between quercetin and copper ions in different conditions *in vitro* demonstrated the prooxidative nature of their interplay. For example, quercetin undergoes a slow autooxidation under physiological pH, producing hydrogen peroxide. In the presence of copper, it is more prone to autooxidation, and accordingly generates more hydrogen peroxide [36]. In the presence of Cu^2+^, phenolic compounds such as quercetin are oxidized to a semiquinone radical. The semioxidized quercetin is not stable and undergoes a second oxidation reaction that yields *ortho*-quinone and superoxide anion (O_2_^•^). The superoxide anion reacts with catalytic Cu^+^ and forms hydrogen peroxide that is further converted into a hydroxyl radical (^•^OH) promoting formation of more ROS [62]. However, even the prooxidant activity of quercetin may stimulate a cellular defence response by activating redox-dependent signal transduction pathways leading to the upregulation of antioxidative enzymes [63]. As suggested by Filipe et al. [61], it is likely that prooxidative and antioxidative mechanisms are operative simultaneously, but at certain combinations of copper and quercetin concentrations, one of the mechanisms prevails, determining the overall effect on neuronal viability.

The main oxidized metabolite of quercetin, quercetin-quinone, exerts specific reactivity towards thiol groups which may lead to the loss of protein functions and jeopardize neuronal functioning [64]. At first, quercetin preferentially reacts with GSH; however, at depleted GSH levels, it reacts with protein thiols, forming relatively stable adducts [64]. In our study, the overall effect on GSH content was not consistent with neuronal survival. Quercetin applied at a concentration of 150 *μ*M exacerbated the copper-induced decrease of the GSH content in both mild and severe oxidative stress. GSH levels were similar in neurons exposed to 1 mM CuSO_4_ alone (viability of 35% in comparison to the control group) and in neurons exposed to 0.5 mM CuSO_4_ and 150 *μ*M quercetin (viability of 88% in comparison to the control group). This indicates that downstream effects of ROS on neuronal death pathways and signalling cascades, rather than protein dysfunctions due to the formation of thiol adducts, determine the overall impact on neuronal viability.

Upregulation of ROS levels is usually accompanied with the activation of executioner caspases-3/7, particularly in a mildly oxidative environment [65]. In severe oxidative injury characterized by a prominent decrease in neuronal viability, neuronal cells also die by necrosis [25]. Our study shows that copper induces caspase-3/7 activation in a dose-dependent manner. Treatment with 0.5 mM CuSO_4_ induced a very small (27%), statistically significant increase, whereas effects of 1 mM CuSO_4_ were more than doubled. Structural changes of chromatin are much more pronounced than the effect on caspase-3/7 activity with exposure to CuSO_4_. As condensed nuclei are a hallmark of both caspase-dependent and caspase-independent cell death, we suggest that in mild oxidative stress, neuronal cells predominantly died by a caspase-independent mechanism, but that in a highly oxidative environment, the classic apoptotic machinery is triggered, together with necrotic death, as the number of dead cells exceeded the number of dead cells with chromatin changes [40]. Nevertheless, effects of quercetin on copper-induced enzymatic and nuclear changes were in a good correlation with the overall effect of quercetin on neuronal viability.

Transcription factor p53 may play an important role in determining cell death or survival by guiding cellular response to diverse genotoxic challenges, including oxidative stress, primarily by regulating transcription of various pro- and antiapoptotic genes [43]. Bax is another common effector of cell death. In response to various stress stimuli, it translocates to the mitochondria and induces membrane permeabilization, enabling the release of various proapoptotic effectors [66]. However, in our conditions, the levels of p53 and Bax remained stable. Previous studies have shown that Bax translocation, rather than its upregulation, could be the crucial event for initiating neuronal death after exposure to various agents that increase production of ROS [67]. As for Bax, it is possible that p53 translocation, instead of p53 upregulation, is the most important factor for the initiation of death events at this concentration of copper [68, 69]. However, quercetin also did not modify expression of either p53 or Bax indicating that these proteins did not contribute to the prosurvival effect of quercetin.

PUMA is a potent inducer of p53-independent death. It is able to activate Bax [70] and antagonize the function of antiapoptotic members of the Bcl-2 family [71]. Under basal conditions, PUMA is expressed in very small amounts, but is rapidly induced by various forms of stressors, including altered redox status. Its expression could be regulated by various transcription factors that respond to toxic stimuli [71]. The PUMA gene promoter contains binding sites for several transcription factors in addition to p53 (such as p73, c-Myc, FoxO3A, CHOP, E2F1, and AP-1), which could be differentially activated in different forms of cellular stress [72]. This probably may explain our results showing prominent copper-induced upregulation of PUMA together with unchanged p53 levels. Cotreatment with quercetin partially prevented PUMA induction, indicating that the suppression of PUMA activation could be the underlying mechanism of the observed neuroprotective action of quercetin in P19 neurons.

The role of NME1 in oxidative stress response has been recently emerging. This enzyme is mostly known for its nucleoside diphosphate kinase activity, although it also may exert many functions within the cell nucleus as well. Recent data indicate that DNA-damaging agents induce the presence of NME1 in the nucleus where it may regulate gene transcription acting as a transcription factor, or it may participate in the repair of DNA strand breaks [44]. Furthermore, NME1 overexpression promoted the survival of primary rat cortical neurons during excitotoxicity, hydrogen peroxide-induced oxidative stress, and oxygen-glucose deprivation [73]. NME1 was also secreted extracellularly in an *in vitro* model of traumatic brain injury which also might indicate its neuroprotective and regenerative function [74]. In line with these findings, NME1 expression was upregulated in quercetin-treated P19 neurons, suggesting its important role in promoting neuronal survival in copper-induced oxidative stress. Of note, it has been shown that NME1 may modulate Akt and ERK signalling pathways [75].

**Figure 7:**
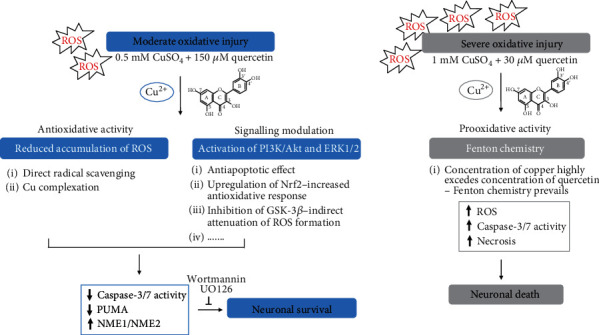
Schematic representation of copper/quercetin interactions in P19 neurons. Depending on the severity of copper-induced oxidative injury and concentration of quercetin used, both prosurvival and prodeath effects were observed because of copper/quercetin interactions. In moderate oxidative injury, 150 *μ*M quercetin promotes neuronal survival by reducing the production of ROS and through the activation of the PI3K/Akt and ERK1/2 signalling pathways. We hypothesize that the quercetin-induced activation of these pathways may exert antiapoptotic effects, stimulate an antioxidative response by activating the transcription factor Nrf2, and inhibit the activity of glycogen synthase kinase-3*β* (GSK-3*β*), thus indirectly attenuating ROS formation. The antioxidative effects of quercetin are probably mediated through direct ROS scavenging and Cu complexation which prevents Fenton's chemistry. The antioxidative activity and modulation of intracellular signalling ultimately reduce the death cascade, prevent PUMA upregulation, and stimulate the expression of the NME1 protein. In severe oxidative stress conditions, 30 *μ*M quercetin exacerbated the toxic effect of copper. We suggest that at this concentration of quercetin, ROS formation is promoted due to the ability of quercetin to reduce copper and initiate Fenton's reaction and the production of hydroxyl radicals. An increase in ROS further leads to apoptotic and necrotic changes, and ultimately neuronal death. At 150 *μ*M quercetin, probably more copper ions are complexed, thus holding the initiation of Fenton's chemistry, and more quercetin molecules are available to act as ROS scavengers. However, as both copper and quercetin are present at relatively high concentrations, the sum of all antioxidative and prooxidative activities (depicted in Figure 1) in the end does not affect neuronal survival.

Growing evidence indicates that flavonoids exert neuroprotective effects by modulating various signalling cascades [18, 19]. We have previously shown that quercetin promotes the survival of P19 neurons in hydrogen peroxide-induced oxidative stress by modulating Akt and ERK1/2 signalling [26]. Similarly, pharmacological inhibition of PI3K/Akt and ERK1/2 pathways prevents the beneficial effects of quercetin on copper-induced neuronal death. Signal transduction through the PI3K/Akt and ERK1/2 pathways contributes to many aspects of neuronal functioning, and both pathways are considered as potential therapeutic targets in neurodegeneration [76, 77]. It has been shown that quercetin may induce the expression of the brain-derived neurotrophic factor (BDNF) which in turn activates the ERK and PI3K pathways [78]. Potential targets, as well as underlying mechanisms, that possibly determine the net survival of P19 neurons following exposure to quercetin and copper, are represented in Figure 7.

## 5. Conclusions

We observed a neuroprotective effect at a particular concentration of quercetin in modest oxidative stress conditions induced by exposure to divalent copper ions. In addition to antioxidative activities, this effect is mediated through the Akt and ERK signalling cascades. Quercetin reduces the increase in PUMA expression and upregulates NME1, which ultimately prevents the stimulation of caspase-3/7 activity and chromatin condensation. As such, quercetin and other flavonoids acting as modulators of Akt and ERK signalling pathways represent promising therapeutic approaches to alleviate the consequences of mild copper-induced oxidative stress.

## Data Availability

The data used to support the findings are available upon request from the corresponding author.

## Supplementary Materials

Supplementary Materials Figure S1: differentiation of P19 neuronal cells. Immunofluorescence staining against *β*-III tubulin demonstrates complete neuronal differentiation.
