# Supplementary material for: PI3K/Akt and ERK1/2 Signalling Are Involved in Quercetin-Mediated Neuroprotection against Copper-Induced Injury
Source: Oxid Med Cell Longev. 2020 Jul 11;2020:9834742. doi: 10.1155/2020/9834742 (PMC7369662; doi:10.1155/2020/9834742)
Supplement: Supplementary Materials — Figure S1: differentiation of P19 neuronal cells. Immunofluorescence staining against β-III tubulin demonstrates complete neuronal differentiation. [file 9834742.f1.docx]

**Supplementary material**


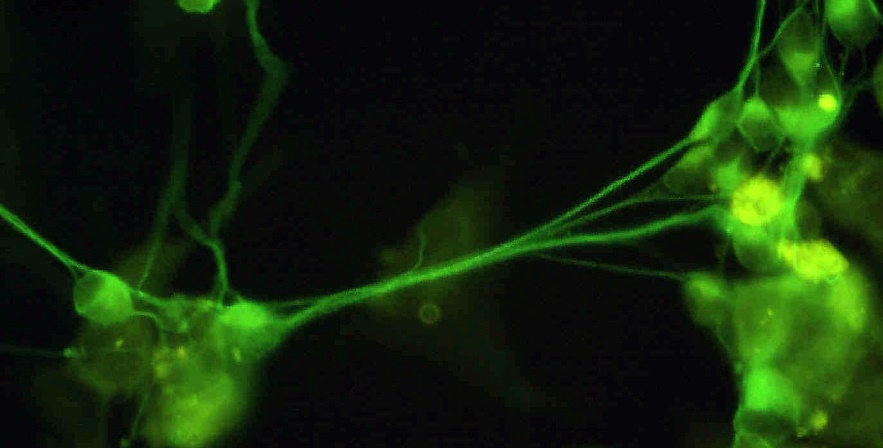


Figure S1. Differentiation of P19 neuronal cells. Immunofluoresence staining against β-III tubulin demonstrates complete neuronal differentiation.
